# Supplementary material for: Interpolation and differentiation of alchemical degrees of freedom in machine learning interatomic potentials
Source: Nat Commun. 2025 May 10;16:4350. doi: 10.1038/s41467-025-59543-2 (PMC12065795; doi:10.1038/s41467-025-59543-2)
Supplement: Supplementary file 1 — Supplementary Information [file 41467_2025_59543_MOESM1_ESM.pdf]

# Supplementary Information for:
# Interpolation and differentiation of alchemical degrees of freedom in machine learning interatomic potentials

## I. INTERPOLATION

### A. Comparison of interpolation schemes

We investigate alternative interpolation schemes for the asymmetric edge weight (equation (5) in the main text) to compare their ability to interpolate the model energy output. For simplicity, we consider interpolating between the two compositional states. First, we can think of a symmetric edge weighting scheme $\omega_{\alpha\beta} = \lambda_\alpha \lambda_\beta$, which also satisfies the consistencies at both ends of the alchemical interpolation where one of the weights is one, and the other is zero. Alternatively, without creating extra alchemical atoms, we can change the atomic identities in the input graph by linear interpolating their atomic embeddings. The alchemical modifications in the main text are retained for the symmetric scheme, and the original MLIP architecture is used for embedding interpolation. We revisit the examples in the main text and interpolate between NaCl–KCl and the $\alpha$ phases of $CsPbI_3$–$CsSnI_3$. We used MACE-MP-0 medium and small models [1], respectively, and evaluated the energy of the system during interpolation, as shown in Fig. S1.

As argued in the main text, since the asymmetric embedding scheme satisfies the consistencies with the original message passing, the resulting energy values are close to the linearly interpolated energy values in both cases. The two consistencies in the main text do not hold when using the symmetric weighting scheme, which causes the energy values to exhibit a significant positive deviation during interpolation. Finally, since discrete elemental embeddings would not lie in an affine subspace, a linear interpolation of atomic embeddings may go through unmeaningful embedding values, and this may cause significant fluctuations (Fig. S1b). Furthermore, the occupancy of the atom should be retained when only changing the embeddings, so we cannot create or annihilate atoms as we did to calculate the free energy of vacancy.

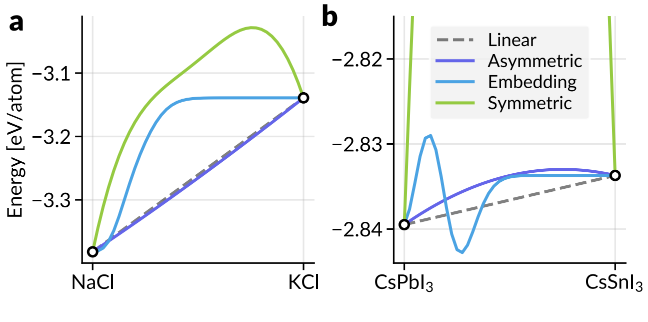


FIG. S1. **Comparison of various interpolation schemes.** Energy values obtained from different interpolation schemes: asymmetric weighting (as in the main text), linear interpolation of atomic embeddings, and symmetric weighting, for the interpolation between (a) NaCl–KCl and (b) the $\alpha$ phases of $CsPbI_3$–$CsSnI_3$. Source data are provided as a Source Data file.

TABLE SI. **Computational efficiency for different alchemical interpolation schemes.** Computation time per step for the alchemical switching between $\alpha$-$CsPbI_3$ and $CsSnI_3$ structures under the NPT ensemble.

| Supercell size | Number of atoms | Linear [ms/step] | Alchemical [ms/step] | Speedup |
|---|---|---|---|---|
| $3 \times 3 \times 3$ | 135 | 36 | 20 | 1.80 |
| $4 \times 4 \times 4$ | 320 | 45 | 28 | 1.62 |
| $5 \times 5 \times 5$ | 625 | 83 | 48 | 1.73 |
| $6 \times 6 \times 6$ | 1,080 | 120 | 76 | 1.58 |

### B. Alchemical vs. linear interpolation of energies

Here, we compare the proposed alchemical interpolation scheme with a naive linear interpolation of endpoint energies. While evaluating the linear interpolation would require the evaluation of two input graphs corresponding to both endpoints, we are introducing extra alchemical atoms and doing backpropagation to obtain alchemical gradients in the alchemical modification scheme. We benchmarked both interpolation schemes on the alchemical pathway between $\alpha$-phase structures of $CsPbI_3$ and $CsSnI_3$, as in the main text. For the alchemical scheme, we are adding 20% more nodes (Pb or Sn) to the input graph and evaluate the gradient with respect to three variables (positions, strains, and alchemical weights); for the linear scheme, we evaluate the gradient with respect to two variables (positions and strains), but we compute them twice (two endpoints) per each MD step. The time taken per step for each scheme for various supercell sizes using a single NVIDIA Volta V100 32GB GPU with 20 cores of an Intel Xeon Gold 6248 CPU are reported in Table SI.

The results show that the alchemical scheme is more efficient than the linear scheme, offering 1.6 to 1.8 times the speedup. This is because most of the backward pass for the alchemical gradient coincides with that of the forces and stress. The extra overhead of backpropagation to the alchemical weights would be relatively small. Furthermore, if three or more alchemical states were considered [2, 3], the alchemical scheme would be even more relatively efficient.

Now, we assess whether the two interpolation schemes offer comparable alchemical free energy calculation re-

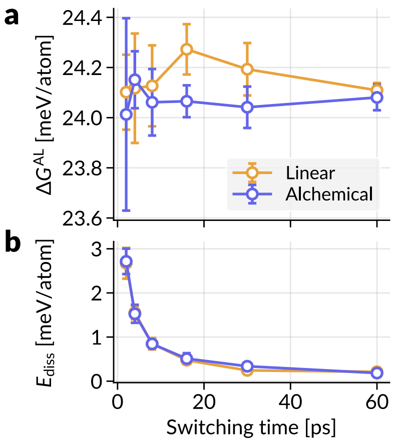


FIG. S2. **Alchemical free energy simulation results from different alchemical interpolation schemes.** (a) The alchemical free energy difference $\Delta G^{\mathrm{AL}}$ of Pb $\rightarrow$ Sn conversion in $\alpha$ phase at 400 K and 1 atm and (b) the average dissipated energies (equation (10) in the main text) plotted against the switching time. Each data point represents the average of four statistically independent simulations, with standard deviations shown as error bars. Source data are provided as a Source Data file.

sults. We follow the same simulation procedure used to produce Fig. 6c in the main text, while using the energy difference between two endpoint Hamiltonians as the alchemical gradient in the linear scheme case: $\partial H/\partial\lambda = H_\mathrm{f} - H_\mathrm{i}$. First, when the switching time is long enough, the alchemical free energy differences obtained from the linear and alchemical methods converge to the same value. Furthermore, not much difference is observed in the convergence of $\Delta G^{\mathrm{AL}}$ or the trend of dissipated energies as the switching time increases. Hence, the alchemical scheme developed in this work could be regarded as a computationally efficient alternative to naive linear interpolation of endpoint energies in the context of free energy calculations.

## II. CHGNET RESULTS

### A. Alchemical modifications

We outline how the alchemical modifications could be made to the CHGNet model [4]. In CHGNet, the node and edge features are updated as in equations (1) and (2) (equation (2) in the original paper). The modification to message passing in equation (6) in the main text is implemented by applying the weights $\omega_{\alpha\beta}$ (equation (5) in the main text) to the summands of the message aggregation as in equations (3) and (4):

$$\boldsymbol{v}_i^{t+1} = \boldsymbol{v}_i^t + L_\mathrm{v}^t \left[ \sum_j \boldsymbol{e}_{ij}^\mathrm{a} \odot \phi_\mathrm{v}^t \left( \boldsymbol{v}_i^t \| \boldsymbol{v}_j^t \| \boldsymbol{e}_{ij}^t \right) \right], \tag{1}$$

$$\boldsymbol{e}_{jk}^{t+1} = \boldsymbol{e}_{jk}^t + L_\mathrm{e}^t \left[ \sum_i \boldsymbol{e}_{ij}^\mathrm{b} \odot \boldsymbol{e}_{jk}^\mathrm{b} \odot \phi_\mathrm{e}^t \left( \boldsymbol{e}_{ij}^t \| \boldsymbol{e}_{jk}^t \| \boldsymbol{a}_{ijk}^t \| \boldsymbol{v}_j^{t+1} \right) \right], \tag{2}$$

$$\boldsymbol{v}_{(i,\alpha)}^{t+1} = \boldsymbol{v}_{(i,\alpha)}^t + L_\mathrm{v}^t \left[ \sum_{(j,\beta)} \omega_{\alpha\beta} \boldsymbol{e}_{ij}^\mathrm{a} \odot \phi_\mathrm{v}^t \left( \boldsymbol{v}_{(i,\alpha)}^t \| \boldsymbol{v}_{(j,\beta)}^t \| \boldsymbol{e}_{(i,\alpha),(j,\beta)}^t \right) \right], \tag{3}$$

$$\boldsymbol{e}_{(j,\beta),(k,\gamma)}^{t+1} = \boldsymbol{e}_{(j,\beta),(k,\gamma)}^t + L_\mathrm{e}^t \left[ \sum_{(i,\alpha)} \omega_{\beta\alpha} \boldsymbol{e}_{ij}^\mathrm{b} \odot \boldsymbol{e}_{jk}^\mathrm{b} \odot \phi_\mathrm{e}^t \left( \boldsymbol{e}_{(i,\alpha),(j,\beta)}^t \| \boldsymbol{e}_{(j,\beta),(k,\gamma)}^t \| \boldsymbol{a}_{(i,\alpha),(j,\beta),(k,\gamma)}^t \| \boldsymbol{v}_{(j,\beta)}^{t+1} \right) \right]. \tag{4}$$

Instead of defining angle weights, we can also use $\omega_{\beta\alpha}$ (equation (5) in the main text) for the bond feature updates, considering that the weight of the message sender should only be taken into account when the message contributions from alchemical objects are being aggregated.

For the original readout in equation (5) (equation (5) in the original paper [4]), we implement the alchemical readout (equation (7) in the main text) as in equation (6):

$$E_\mathrm{tot} = \sum_i L_3 \circ g \circ L_2 \circ g \circ L_1 \left( v_i^4 \right), \tag{5}$$

$$E_\mathrm{tot} = \sum_{(i,\alpha)} \lambda_\alpha L_3 \circ g \circ L_2 \circ g \circ L_1 \left( v_{(i,\alpha)}^4 \right). \tag{6}$$

Although a similar modification could be made, this scheme is not as efficient as in the MACE model because the CHGNet model introduces extra features on bonds and angles for the message passing. This introduces tensors containing multiple alchemical indices, and

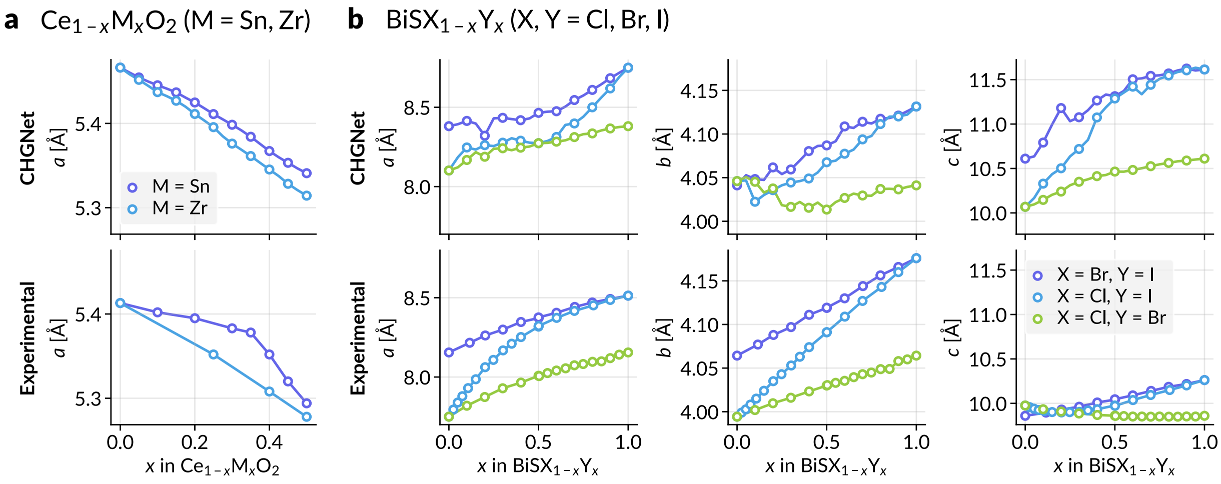


FIG. S3. **Lattice parameters for solid solutions using the CHGNet model.** (a) Lattice parameter $a$ for $Ce_{1-x}M_xO_2$ (M = Zr, Sn) as a function of the compositional weight $x$. (b) Lattice parameters $a$, $b$, and $c$ for $BiSX_{1-x}Y_x$ (X, Y = Cl, Br, I) as a function of $x$. The upper panels are the result of the alchemically modified CHGNet model [4], and the lower panels are the experimental result from [5] and [6] for (a) and (b), respectively. Source data are provided as a Source Data file.

the overhead from the alchemical modification becomes more significant.

### B. Lattice parameters

To benchmark the alchemically modified CHGNet model on determining the cell parameters of solid solutions, we conducted the same calculations as in Fig. 2 in the main text. The trend in Fig. S3 is more rugged than the results from the MACE model, possibly due to the extra bond and angle features updated on each message passing step. Furthermore, the cell parameters for pure compounds do not match their experimental values well in this case. We note that the exceptions to the Vegard's law explored in the current work are peculiar cases and would not mean that the baseline performance of CHGNet is worse than MACE in general. However, it still reveals that the proposed alchemical scheme in this work could be integrated much more nicely with the universal MACE model.

## III. ALCHEMICAL FREE ENERGY CALCULATION

Here, we examine the impact of phase transformation on the discrepancy between the Frenkel–Ladd and alchemical paths, as shown in Fig. 6 of the main text. Each free energy simulation begins from the energy-minimized structure of $\alpha$-$CsPbI_3$. In the Frenkel–Ladd path (NVT), we equilibrate the system using a homogeneous Berendsen thermostat, which maintains the relative scales of the cell parameters, while the alchemical path (NPT) is equilibrated using an inhomogeneous Berendsen thermostat. We track the evolution of cell parameters during

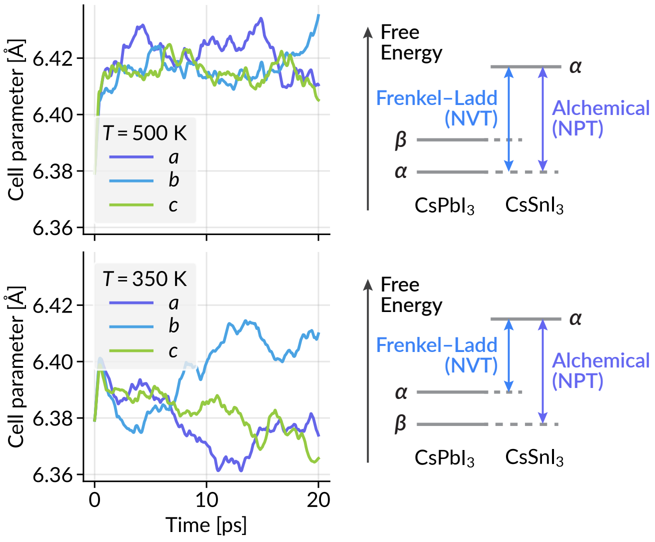


FIG. S4. **Equilibrium cell parameters and free energies for perovskite phases.** (Left) Evolution of cell parameters $a$, $b$, and $c$ for perovskite $CsPbI_3$ structures at 500 K and 350 K during NPT equilibration. (Right) Schematic of corresponding free energy profiles at each temperature. Equilibrium cell parameters of $CsPbI_3$ reveal the lowest free energy state between perovskite phases $\alpha$ and $\beta$, highlighting differences between free energies calculated via the Frenkel–Ladd path (NVT) and the alchemical path (NPT). Source data are provided as a Source Data file.

alchemical path equilibration in Fig. S4 (left) at 500 K and 350 K. At 500 K, all cell parameters remain consistent (cubic, $\alpha$), while at 350 K, they equilibrated into a tetragonal structure with $a \approx c < b$, indicating an initial transition from $\alpha$ to $\beta$. Since the structure at $CsSnI_3$ composition remains in the $\alpha$ (cubic) phase, as shown in

the free energy schematic (Fig. S4, right), the lower free energy perovskite phase ($\beta$) of $CsPbI_3$ leads to a larger free energy difference for the alchemical path.

[1] I. Batatia, P. Benner, Y. Chiang, A. M. Elena, D. P. Kovács, J. Riebesell, X. R. Advincula, M. Asta, M. Avaylon, W. J. Baldwin, F. Berger, N. Bernstein, A. Bhowmik, S. M. Blau, V. Cărare, J. P. Darby, S. De, F. D. Pia, V. L. Deringer, R. Elijošius, Z. El-Machachi, F. Falcioni, E. Fako, A. C. Ferrari, A. Genreith-Schriever, J. George, R. E. A. Goodall, C. P. Grey, P. Grigorev, S. Han, W. Handley, H. H. Heenen, K. Hermansson, C. Holm, J. Jaafar, S. Hofmann, K. S. Jakob, H. Jung, V. Kapil, A. D. Kaplan, N. Karimitari, J. R. Kermode, N. Kroupa, J. Kullgren, M. C. Kuner, D. Kuryla, G. Liepuoniute, J. T. Margraf, I.-B. Magdău, A. Michaelides, J. H. Moore, A. A. Naik, S. P. Niblett, S. W. Norwood, N. O'Neill, C. Ortner, K. A. Persson, K. Reuter, A. S. Rosen, L. L. Schaaf, C. Schran, B. X. Shi, E. Sivonxay, T. K. Stenczel, V. Svahn, C. Sutton, T. D. Swinburne, J. Tilly, C. van der Oord, E. Varga-Umbrich, T. Vegge, M. Vondrák, Y. Wang, W. C. Witt, F. Zills, and G. Csányi, A foundation model for atomistic materials chemistry (2024), arXiv:2401.00096 [physics.chem-ph].

[2] J. L. Knight and C. L. Brooks III, Multisite $\lambda$ dynamics for simulated structure–activity relationship studies, J. Chem. Theory Comput. **7**, 2728 (2011).

[3] M. T. Robo, R. L. Hayes, X. Ding, B. Pulawski, and J. Z. Vilseck, Fast free energy estimates from $\lambda$-dynamics with bias-updated Gibbs sampling, Nat. Commun. **14**, 8515 (2023).

[4] B. Deng, P. Zhong, K. Jun, J. Riebesell, K. Han, C. J. Bartel, and G. Ceder, CHGNet as a pretrained universal neural network potential for charge-informed atomistic modelling, Nat. Mach. Intell. **5**, 1031 (2023).

[5] T. Baidya, P. Bera, O. Kröcher, O. Safonova, P. M. Abdala, B. Gerke, R. Pöttgen, K. R. Priolkar, and T. K. Mandal, Understanding the anomalous behavior of Vegard's law in $Ce_{1-x}M_xO_2$ (M = Sn and Ti; $0 < x \leq 0.5$) solid solutions, Phys. Chem. Chem. Phys. **18**, 13974 (2016).

[6] P. Schultz and E. Keller, Strong positive and negative deviations from Vegard's rule: X-ray powder investigations of the three quasi-binary phase systems $BiSX_{1-x}Y_x$ (X, Y = Cl, Br, I), Acta Cryst. B **70**, 372 (2014).
